# Stevens-Johnson syndrome-toxic epidermal necrolysis spectrum reactions to immune checkpoint inhibitor therapy and safety of rechallenge: A retrospective review

**DOI:** 10.1016/j.jdcr.2025.03.039

**Published:** 2025-06-10

**Authors:** Gunnar Mattson, Rodrigo Gutierrez, Anna Haemel, Kanade Shinkai, Ryan Arakaki, Lindy Fox, Allison Dobry

**Affiliations:** a School of Medicine, University of California, San Francisco, California; b Department of Dermatology, University of California, San Francisco, California

**Keywords:** drug reaction, immune checkpoint inhibitors, lichenoid dermatitis, SJS, SJS-TEN, TEN

Stevens-Johnson syndrome (SJS), toxic epidermal necrolysis (TEN), and lichenoid dermatitis (LD) represent a spectrum of mucocutaneous eruptions that can occur from immune checkpoint inhibitors (ICIs).^1^ As immunotherapy alters the immune state, associated dermatologic adverse events can be more variable and unpredictable than standard drug eruptions.^1^ These eruptions are thought to be T cell-mediated due to immune dysregulation whereby the drug binds to T-cell and major histocompatibility complex class I receptors, inducing replication of CD8+ T-cells that attack keratinocytes.^1^^,^^2^ This hypothesis is supported by biopsy sections of ICI-induced SJS, which have shown increased CD8+ T-cells in the dermal-epidermal junction and increased PD-L1 expression in keratinocytes.^3^ Unfortunately, stopping immunotherapy has profound implications on oncologic therapeutic options and survival, making it difficult to determine treatment course and re-exposure safety.^4^ This retrospective observational study aims to characterize clinical outcomes of SJS-TEN spectrum eruptions to ICIs and evaluate rechallenge safety. The University of California San Francisco dermatology inpatient log was queried for cases of SJS/TEN/LD from 2009 to 2022, yielding 34 unique episodes, including 10 (29%) attributed to ICIs.

The most implicated ICI agent was pembrolizumab, followed by nivolumab, then ipilimumab. Mean patient age was 61.4 years and 50% were male. Six patients had severe LD, 3 had SJS, and one had TEN. Mean ICI therapy duration was 97.9 days (range 14-420 days), and mean time between last dose and rash onset was 38.5 days. One patient had peripheral eosinophilia, 4 had fever at rash onset, and 6 had mucosal involvement. Suspected agents were stopped in all patients. Nine patients were treated with oral prednisone, with 2 also receiving IVIg. The remaining patient received only topical steroids. All 10 patients survived the reaction, and 4 patients (one with SJS) were rechallenged with the same ICI agent after discussion with oncology. The SJS patient was rechallenged with nivolumab while withholding his indoleamine 2,3-dioxygenase inhibitor. Two patients (including the SJS patient) tolerated rechallenge without rash recurrence, 1 patient experienced a mild rash that responded to oral steroids and continued immunotherapy, and 1 patient suffered another severe reaction warranting repeated withdrawal and treatment. On histologic analysis, 9 patients displayed interface dermatitis [(vacuolar (*n* = 4), lichenoid(*n* = 2), mixed vacuolar, and lichenoid(*n* = 3)], 2 showed full-thickness necrosis, and 5 demonstrated eosinophils (Table I).

**Table I:** Summary of culprit immune checkpoint inhibitor agent identified, histopathology, symptoms at eruption onset, and rechallenge outcome (if attempted)

Clinical course	Outcome
Culprit ICI agent	n = 11 (100%)
Pembrolizumab	8 (80%)
Nivolumab	2 (20%)
Ipilimumab	1 (10%)
Histopathology	n = 10 (100.0%)
Vacuolar dermatitis	7 (70%)
Lichenoid dermatitis	5 (50%)
Full-thickness necrosis	2 (20%)
Presence of eosinophils	50%
Symptom	n = 10 (100.0%)
Fever	4 (40%)
Mucous membrane involvement	6 (60%)
Peripheral eosinophilia	1 (10%)
Rechallenge outcome	
Tolerated without treatment	2 (20%)
Tolerated with oral steroids	1 (10%)
Not tolerated	1 (10%)
Rechallenge not attempted	6 (60%)

*ICI*, Immune checkpoint inhibitor.

This study highlights the spectrum of SJS-TEN drug eruptions secondary to ICI therapy and rechallenge safety outcomes. One patient with SJS successfully tolerated rechallenge, and thus his presentation may be more consistent with progressive immunotherapy-related mucocutaneous eruption.^5^ The variable treatment duration prior to eruption and clinical course following rechallenge demonstrates the need for further studies evaluating the immunologic underpinnings of these ICI eruptions and the unique histopathologic and cytokine markers that distinguish ICI-induced SJS-TEN from other forms in order to optimize diagnosis and treatment guidelines in these complex oncodermatologic eruptions. This study is limited by single-center design, small study population, and retrospective nature. Future studies involving larger cohorts, additional health care centers, and more prospective studies may identify additional factors that inform the safety of rechallenging patients to ICI agents.

## Conflicts of interest

None disclosed.
